# Interplay Between Thyrotroph Morphometry and Circulating Thyroid Hormones in Lactating and Non-Lactating Female Camels (*Camelus dromedarius*)

**DOI:** 10.3390/vetsci12090917

**Published:** 2025-09-22

**Authors:** Shaukat Ali Shaukat Jaspal, Muhammad Mubashar Shaukat, Robina Shaukat, Tahmina Shaukat, Abdul Majeed Cheema, Rifat Ullah Khan, Shabana Naz, Babar Maqbool, Caterina Losacco, Naila Chand, Ibrahim A. Alhidary

**Affiliations:** 1 Institute of Zoology, University of the Punjab, Lahore 25100, Pakistan; 2 Department of Zoology, Wildlife and Fisheries, University of Agriculture, Faisalabad 38000, Pakistan; 3 Physiology Lab, Faculty of Animal Husbandry & Veterinary Sciences, College of Veterinary Sciences, The University of Agriculture, Peshawar 25130, Pakistan; 4 Department of Zoology, Government College University, Faisalabad 38000, Pakistan; 5 Department of Veterinary Medicine, Faculty of Veterinary & Animal Sciences, The University of Agriculture, Dera Ismail Khan 29163, Pakistan; 6 Department of Precision and Regenerative Medicine and Jonian Area (DiMePRe-J), Section of Veterinary Science and Animal Production, University of Bari ‘Aldo Moro’, s.p. Casamassima km 3, 70010 Valenzano, Italy; 7 Department of Poultry Science, Faculty of Animal Husbandry & Veterinary Sciences, The University of Agriculture, Peshawar 25130, Pakistan; 8 Department of Animal Production, College of Food and Agriculture Science, King Saud University, Riyadh 11451, Saudi Arabia

**Keywords:** dromedary camel, thyrotrophs, thyroid hormones, lactation, age

## Abstract

This study investigated the morphometric characteristics of adenohypophyseal thyrotrophs and circulating thyroid hormone profiles in dromedary camels (*Camelus dromedarius*) in relation to age and lactation status. Clinically healthy Brela breed camels were divided into lactating female, and non-lactating female groups across two age categories (5–10 years and ≥11 years), with fifty animals per group. Blood samples were collected before slaughter and pituitary glands were collected post-slaughter and processed for immunohistochemical detection of thyroid-stimulating hormone (TSH) using anti-porcine TSHβ antibody, while morphometric measurements of thyrotrophs were conducted through image analysis. Plasma concentrations of TSH, triiodothyronine (T_3_), and thyroxine (T_4_) were quantified using validated ELISA and enzyme immunoassay kits. Group differences were analyzed using one-way ANOVA followed by post hoc comparisons, with statistical significance set at *p* < 0.05. Morphometric analysis revealed that lactating female camels exhibited significantly higher thyrotroph counts compared with non-lactating counterparts, whereas non-lactating females displayed larger cell and nuclear dimensions. Age influenced these patterns, with older camels showing hypertrophied thyrotrophs but reduced functional plasticity compared to younger animals. Plasma hormone assays demonstrated that non-lactating camels had higher TSH and T_4_ concentrations, while lactating camels maintained elevated T_3_ levels, suggesting enhanced peripheral conversion of T_4_ to T_3_ during milk production. Additionally, younger camels exhibited higher T_3_ concentrations than older animals, indicating age-related decline in thyroidal activity. These findings highlight the dynamic regulation of the hypothalamic–pituitary–thyroid axis in camels, demonstrating how lactation and age shape thyroidal morphology and function to meet diverse physiological demands. These findings not only broaden the comparative endocrinology of underexplored species but also provide physiopathological insights relevant to farm animal management, lactation efficiency, and adaptive metabolism in harsh environments.

## Simple Summary

This study explored the effect of lactation on thyroid activity in dromedary camels. We examined hormone levels in the blood and the cells in the pituitary gland that regulate thyroid function. The results showed that lactating females had more thyroid-regulating cells, while non-lactating females had larger cells and higher levels of certain hormones. Younger camels had higher levels of active thyroid hormone than older ones, suggesting a decline in thyroid activity with age. Overall, these findings show that camels adjust their thyroid function depending on age and lactation needs, especially to support milk production. This work provides new knowledge about camel biology and offers insights that may be useful for farm management, animal health, and understanding how animals adapt to harsh environments.

## 1. Introduction

The dromedary camel (*Camelus dromedarius*) is a vital domestic animal in arid and semi-arid regions, serving as a source of milk, meat, transportation, and economic stability for millions [1,2]. Globally, the population of dromedary camels exceeds 35 million, with the majority concentrated in North Africa, the Middle East, and South Asia [3,4,5]. In Pakistan alone, approximately one million camels are reared, of which the Brela breed is particularly valued for its dual-purpose milk and meat production [6]. Brela camels are predominantly distributed in the Cholistan desert and adjoining arid zones of Punjab, where they are managed under extensive grazing systems with limited feed and water availability [7]. Unlike other livestock, camels have evolved exceptional adaptations to extreme desert environments marked by heat stress, water scarcity, and limited feed availability [8,9]. These adaptations are governed not only by behavioral and physiological mechanisms but also by distinct endocrine regulatory systems [10,11]. Among these, the endocrine control in lactating camels is particularly important in regulating energy metabolism, milk production, and reproductive success under challenging environmental conditions [10]. Understanding these mechanisms is not only relevant for camel production systems but also has wider comparative implications, as it may inform physiopathological insights across farm animal species and contribute to veterinary endocrinology and comparative medicine.

The hypothalamic–pituitary–thyroid (HPT) axis is central to metabolic homeostasis in mammals [11]. Thyroid-stimulating hormone (TSH), produced by thyrotrophs in the anterior pituitary (adenohypophysis), stimulates thyroid hormone secretion, influencing thermoregulation, basal metabolic rate, and reproductive functions [12]. Changes in the structure and size of thyrotrophs often reflect functional alterations of the HPT axis during various physiological states, such as reproduction, aging, and lactation [13]. Despite the critical role of this axis, morphometric studies on pituitary thyrotrophs in camels remain unexplored.

Lactation, one of the most metabolically demanding states, requires dynamic hormonal adjustments to support milk synthesis and energy redistribution [14]. In dairy species, lactation is associated with changes in pituitary cell structure and thyroid hormone levels [15]. Camel milk, with its unique nutritional and medicinal value, is essential for both calf nourishment and human consumption [1]. However, the influence of lactation on the morphology of pituitary thyrotrophs and its relationship with thyroid hormone secretion remains poorly understood in camels. Exploring this relationship has practical value in improving milk production efficiency and understanding endocrine adaptations that may apply to other domestic ruminants under similar physiological demands.

Age also plays a significant role in regulating endocrine function [12]. As animals age, pituitary endocrine cells undergo structural changes that can impact hormone synthesis and secretion [16]. In camels, advancing age has been associated with reduced milk yield, fertility decline, and susceptibility to metabolic disorders [17]. Therefore, assessing how age interacts with lactation to influence pituitary thyrotroph morphology can provide insight into endocrine adaptations in this species.

Morphometric assessment of pituitary cells serves as an indirect measure of their functional state, with larger cellular and nuclear dimensions often indicating heightened secretory activity during physiologically demanding conditions [18]. Immunocytochemical methods for detecting TSH-secreting thyrotrophs allow for precise characterization of their structural attributes. While such studies have been performed in ruminants and poultry [19,20,21], there is a lack of comparable data for camels.

Previous studies in camel endocrinology have primarily addressed circulating thyroid hormone levels or thyroid gland histology [22,23,24], with limited focus on the pituitary origin of regulation. Notably, no comparative study has examined the morphometric features of adenohypophyseal thyrotrophs in lactating versus non-lactating camels across age groups. Given the camel’s adaptive physiology and growing importance in food security under climate stress, this gap in knowledge warrants investigation. By situating camel endocrinology in a broader comparative framework, this study offers insights that extend beyond species-specific physiology, with potential relevance to veterinary endocrinology, livestock productivity, and the understanding of endocrine adaptations under environmental stress.

The present study was therefore designed to evaluate the morphometric characteristics of adenohypophyseal thyrotrophs in lactating and non-lactating female camels (*Camelus dromedarius*) in two age groups (5–10 years and ≥11 years). Using immunocytochemical analysis, thyrotrophs were assessed for cell count, nuclear and cytoplasmic dimensions, and volumetric properties. In view of the findings, which reveal distinct differences in thyrotroph morphology and circulating thyroid hormone concentrations across lactation and age groups, this study provides novel insights into the endocrine adaptations of the dromedary camel. Importantly, these findings underline the practical implications for improving reproductive and productive performance in farm animals, while enriching the comparative endocrinology literature with data from an underexplored species.

## 2. Materials and Methods

### 2.1. Experimental Animals

The present study was conducted on clinically healthy camels (*Camelus dromedarius*) of the Brela breed. Animals were considered clinically healthy based on the absence of visible signs of illness, normal appetite and behavior, and unremarkable findings from routine physical examination (including body condition, rectal temperature, respiratory rate, and heart rate). Anamnestic information provided by owners was also used to exclude animals with a recent history of systemic disease, reproductive disorders, or treatment with hormonal or metabolic drugs. However, we recognize as a limitation that no biochemical analyses of general health biomarkers (e.g., liver and kidney function tests, hematological profiles) were performed, which could have further validated the health status of the animals. The age of the animals was estimated through dental examination and confirmed with information provided by the owners. The study included female dromedary camels, with lactating and non-lactating female camels further categorized into the 5–10 years and ≥11 years age groups. Each group comprised fifty animals. All animals were managed under an extensive grazing system typical of the Cholistan desert, with free access to natural pasture and seasonal browse species, and supplemented with household crop residues when available. Housing was open-air, with animals herded during the day and kept in traditional enclosures at night. Ethical approval for the collection of blood and pituitary tissue samples was obtained from the Institutional Animal Ethics Committee of [Institution name], and procedures were carried out in accordance with national and institutional guidelines for the care and use of animals in research.

### 2.2. Blood Collection

Blood samples (10 mL) were collected from each animal just before slaughtering via direct jugular venipuncture under aseptic conditions to minimize variability due to sampling site. Immediately after collection, samples were transferred into heparinized tubes. The tubes were gently inverted to ensure anticoagulant mixing, then centrifuged at 2500 rpm for 10 min. The resulting plasma was separated and stored at −20 °C until hormone assays were conducted [25].

### 2.3. Sample Collection

Pituitary glands were collected from 50 clinically normal camels using the extirpation technique just after slaughtering by the method described by Jaspal et al. [6]. To prevent hormone diffusion and loss, samples were harvested within four hours post-slaughter.

### 2.4. Tissue Preparation and Immunohistochemistry

Pituitary glands were fixed in Bouin’s solution, dehydrated using standard protocols, and embedded in freshly melted paraffin wax. Midsagittal sections (4 µm thick) were prepared using a rotary microtome (Leica RM-2235) and mounted on Poly-L-Lysine-coated slides (Sigma, St. Louis, MO, USA). Slides were stored in dust-proof boxes for seven days prior to analysis. For immunohistochemistry, slides were immersed in Lugol’s iodine (5 min), followed by 2.5% (*w*/*v*) sodium thiosulphate solution (1 min) to remove residual color. Sections were treated with 3% H_2_O_2_ for 15 min to block endogenous peroxidase activity. After deparaffinization in xylene, sections were incubated in 0.3% H_2_O_2_ in methanol for 30 min. Non-specific binding was blocked using normal goat serum (30 min, room temperature, humidity chamber). Sections were incubated at 37 °C for 120 min with guinea pig–raised anti-porcine TSHβ antibody (dilution 1:50), generously provided by Dr. A.F. Parlow (National Hormone and Peptide Program, NIDDK, Torrance, CA, USA). After washing with Tris-buffered saline (TBS, Sigma, St. Louis, MA, USA, pH 7.4) three times (5 min each), slides were incubated for 30 min with biotinylated goat anti-guinea pig IgG (KPL, Cat. No. 71-00-30). Following another three TBS washes, sections were incubated with a streptavidin–phosphatase complex (KPL, Cat. No. 71-00-45) for 30 min. Immunoreactivity was visualized using Histomark Red solution (KPL, Cat. No. 55-69-00). Finally, sections were rinsed with wash buffer, and excess streptavidin–phosphatase was removed by additional rinsing (5 min). Following the washing steps, tissue sections were treated for 10 min with a working solution of chromogen and DAB substrate (dilution 1:50). The slides were subsequently rinsed, air-dried, and coverslipped using DPX mounting medium. To verify the specificity of the immunostaining, negative control slides were prepared by replacing the primary antibody with 10% normal goat serum. Additional controls were carried out by incubating sections with secondary antibodies raised in a species other than guinea pig, which resulted in no detectable immunoreactivity.

### 2.5. Quantitative Morphometric Assessment

Morphometric analysis was carried out using a compound microscope, considering only thyrotrophs with complete cross-sections and clear, non-reactive nuclei. Six samples per group were examined, with five slides prepared from each sample and ten microscopic fields analyzed per slide. Measurements included thyrotroph count, cell diameter, cell area, cell volume, nucleus diameter, nucleus area, and nucleus volume. Cell and nuclear diameters (µm) were recorded at two perpendicular points (widest axis and 90° angle) using ImageJ software Version 21 (ImageJ (for morphometry) 1.44P, NIH, Bethesda, MD, USA) and AutoCAD 2004 (for volumetric calculations). From these diameters, areas (A = πr^2^) and volumes (V = 4/3 πr^3^) were calculated, following the method of Justin et al. [26].

### 2.6. Hormonal Assays

Plasma thyroid-stimulating hormone (TSH; µIU/mL) was measured using a TSH ELISA kit (Catalog No. DSL-10-5300; Diagnostic Systems Laboratories, Webster, TX, USA). Plasma triiodothyronine (T_3_; ng/mL) was quantified using an Enzyme Immunoassay kit (BioCheck, Burlingame, CA, USA). Total thyroxine was measured using a T_4_ Enzyme Immunoassay kit (BioCheck, Burlingame, CA, USA). All procedures were performed according to the manufacturers’ instructions.

The sensitivity (minimum detectable concentration) of the assays was 0.05 µIU/mL for TSH, 0.2 ng/dL for T_3_, and 0.5 µg/mL for T_4_. The intra-assay coefficients of variation (CVs) for three reference samples (low, medium, and high concentrations) were <6%, <5%, and <7% for TSH; <5%, <6%, and <8% for T_3_; and <6%, <5%, and <7% for T_4_. Inter-assay CVs were consistently below 10% for all assays.

### 2.7. Statistical Analysis

Data were expressed as mean ± standard error of the mean (SEM). Differences among groups were analyzed using one-way analysis of variance (ANOVA), and when significant, means were compared using a post hoc multiple comparison test. All statistical analyses were performed using SPSS software (version 26.0; IBM Corp., Armonk, NY, USA). Statistical significance was set at *p* < 0.05. Means sharing the same superscript letters were considered not significantly different.

## 3. Results

**Figure 1:**
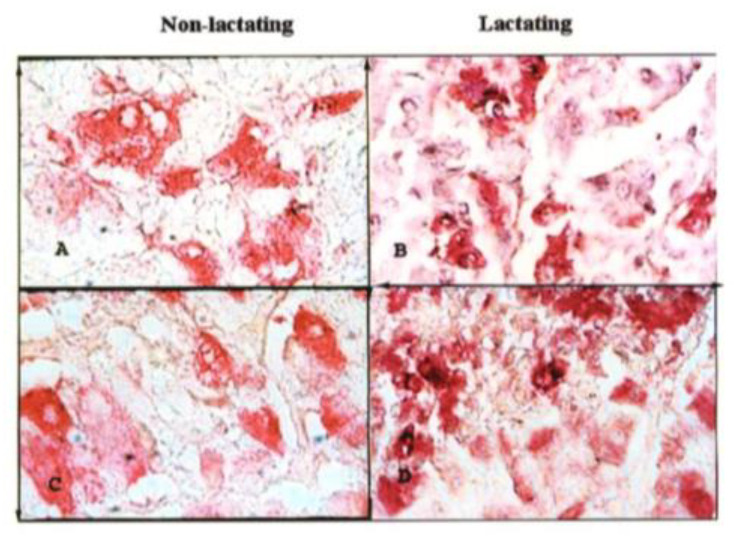
Thyroid-stimulating hormone (TSH) immunoreactive cells in the adenohypophysis of non-lactating and lactating female camels (*Camelus dromedarius*). Streptavidin-alkaline phosphatase immunoreactions: ×5000. (**A**) 5–10 years female (non-lactating): immunoreactive cells appear polygonal with small nuclei. (**B**) 5–10 years female (lactating): immunoreactive cells with small, elongated nuclei. (**C**) 11-onward years female (non-lactating): immunoreactive cells with large elongated with spherical nuclei. (**D**) 11-onward years female (lactating): immunoreactive cells larger than those of young lactating with large nuclei.

The morphometric analysis of adenohypophyseal thyrotrophs in lactating and non-lactating female camels is presented in Table 1 and Figure 1 In the younger age group (5–10 years), non-lactating camels exhibited a significantly higher cellular and nuclear dimensions compared to their lactating counterparts. In contrast, in the older age group (≥11 years), non-lactating camels showed reduced thyrotroph count but larger nuclear dimensions relative to lactating animals. In contrast, in the older age group (≥11 years), lactating camels retained higher thyrotroph counts, whereas non-lactating animals displayed comparatively larger nuclear dimensions. Overall, lactating female camels had significantly higher thyrotroph counts, whereas non-lactating camels demonstrated larger cell and nuclear morphometric parameters.

**Figure 2:**
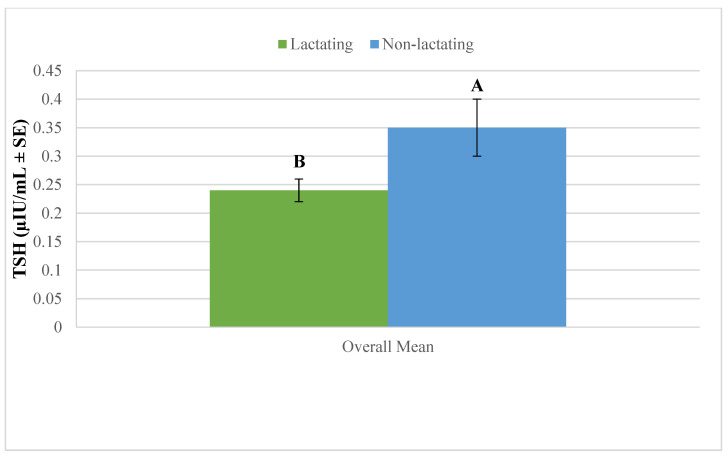
Overall mean plasma thyroid-stimulating hormone (TSH) in lactating and non-lactating camels (*Camelus dromedarius*). Mean values with different superscript letters indicate statistically significant differences at *p* < 0.05.

The overall mean plasma TSH concentration was significantly higher in non-lactating camels compared to their lactating counterparts (Figure 2). This indicates that lactation is associated with a reduction in circulating TSH levels across age groups.

**Figure 3:**
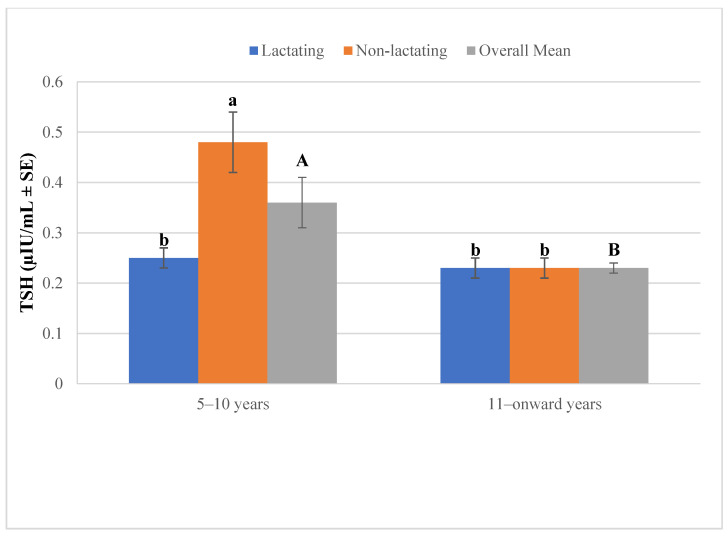
Plasma thyroid-stimulating hormone (TSH) in lactating and non-lactating female camels (*Camelus dromedarius*). Data are expressed as mean ± SEM. Mean values with different superscript letters (uppercase or lowercase) indicate statistically significant differences at *p* < 0.05.

Plasma thyroid stimulating hormone (TSH) concentrations differed significantly between lactating and non-lactating camels, as shown in Figure 3. In the 5–10 years age group, non-lactating animals exhibited markedly higher TSH levels compared to lactating camels, with the overall mean also significantly elevated. In contrast, no significant difference in TSH concentration was observed between lactating and non-lactating camels in the ≥11 years group, where both categories showed consistently lower levels.

**Figure 4:**
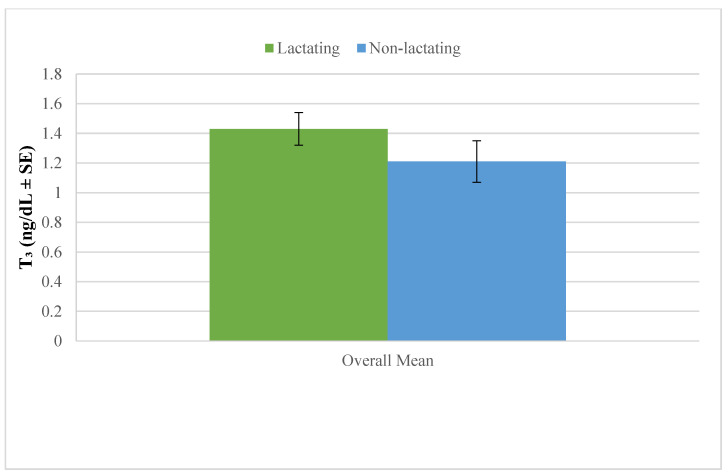
Overall mean plasma triiodothyronine in lactating and non-lactating camels (*Camelus dromedarius*).

Figure 4 shows that the overall mean plasma triiodothyronine (T_3_) concentration was higher in lactating camels compared with non-lactating camels. This indicates that lactation is associated with elevated circulating T_3_ levels in dromedary camels.

**Figure 5:**
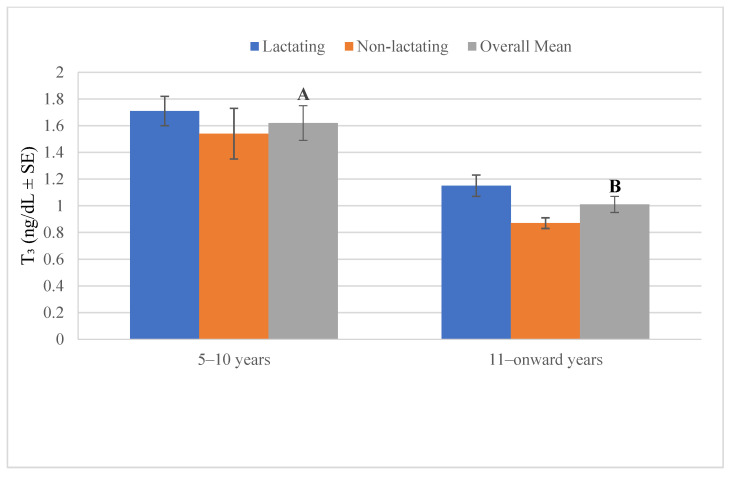
Plasma triiodothyronine in lactating and non-lactating camels (*Camelus dromedarius*). Mean values with different superscript letters indicate statistically significant differences at *p* < 0.05.

Figure 5 shows that plasma triiodothyronine (T_3_) concentrations were higher in camels aged 5–10 years compared with those aged 11 years and above. Within the younger age group, both lactating and non-lactating camels had elevated T_3_ levels, with no marked difference between them, while in the older age group, lactating camels maintained slightly higher T_3_ concentrations than non-lactating ones. The overall mean also reflects a significant decline (A vs. B) in T_3_ levels with advancing age.

**Figure 6:**
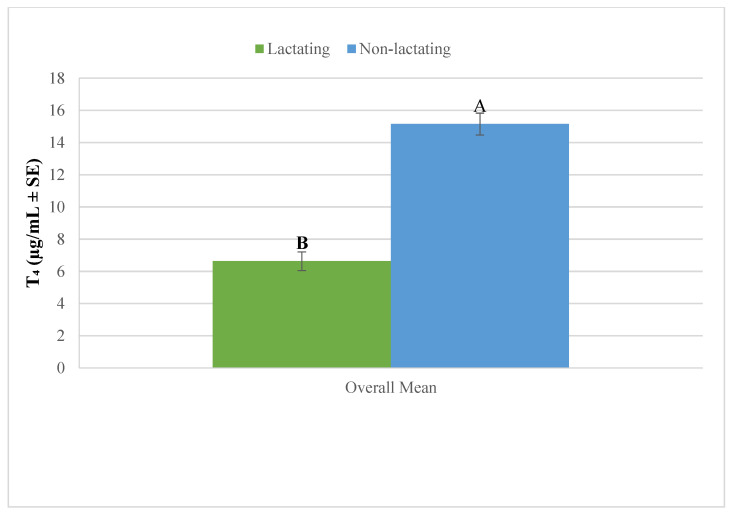
Overall mean plasma thyroxin in lactating and non-lactating camels (*Camelus dromedarius*). Mean values with different superscript letters indicate statistically significant differences at *p* < 0.05.

The results presented in Figure 6 demonstrate that non-lactating camels have significantly higher overall mean plasma thyroxin (T_4_) levels compared to lactating camels (*p* < 0.05). This suggests that the physiological state of lactation is associated with a reduction in plasma T_4_ levels in camels.

**Figure 7:**
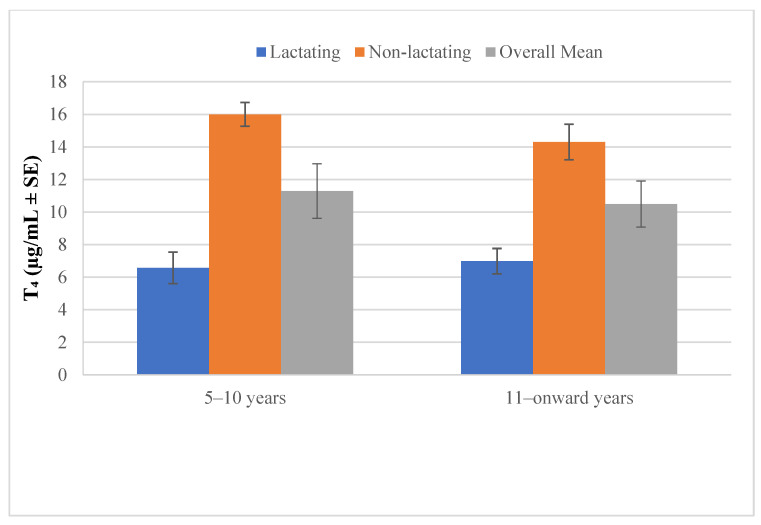
Plasma thyroxin in lactating and non-lactating camels (*Camelus dromedarius*).

Figure 7 shows that plasma thyroxin (T_4_) concentrations were consistently higher in non-lactating camels compared with lactating camels across both age groups. In the 5–10 years group, non-lactating camels exhibited the highest T_4_ levels, while lactating camels had the lowest. A similar trend was observed in camels aged 11 years and above, though with slightly reduced values compared to the younger group. The overall mean also reflects this pattern, indicating that non-lactating camels maintained higher circulating T_4_ levels than lactating ones.

## 4. Discussion

The present study highlights how lactation and age, influence thyrotroph morphology and thyroid hormone profiles in dromedary camels. The results demonstrate the adaptability of the HPT axis to physiological demands such as lactation and aging. Lactation is one of the most metabolically demanding stages in mammals, requiring extensive endocrine adjustments to ensure adequate milk synthesis while maintaining maternal homeostasis [14]. The results of the present study indicate that lactating camels exhibited a higher number of pituitary thyrotrophs, whereas non-lactating camels demonstrated larger thyrotroph cell and nuclear dimensions. Morphologically, an increase in cell count may reflect proliferative adaptation or enhanced turnover of TSH-producing cells, while enlarged nuclear and cytoplasmic areas in non-lactating animals may suggest storage or accumulation of secretory products. Similar adaptations have been reported in other species, where lactation is associated with structural remodeling of anterior pituitary cell populations [27,28].

The endocrine correlates of these morphological changes provide further insights. Plasma TSH concentrations were lower in lactating compared with non-lactating camels, yet immunocytochemical analysis revealed an abundance of thyrotrophs in lactating animals. This paradox may be explained by an increased secretory turnover of TSH during lactation. Consequently, less hormone is retained within cells, leading to lower circulating levels. A comparable pattern was noted in lactating rats, where Ni et al. observed an initial upregulation of hypothalamic pro-TRH mRNA followed by a subsequent decline [18]. These findings are in agreement with the current observations, where lactation appears to be accompanied by an initial stimulatory drive of the HPT axis, followed by adjustments that prioritize efficient thyroid hormone utilization rather than high systemic TSH levels.

Interestingly, lactating camels displayed higher circulating T_3_ levels despite reduced TSH and T_4_. This pattern strongly suggests an enhanced peripheral conversion of T_4_ to T_3_ via deiodinase activity, an adaptive mechanism widely described in mammals during lactation [18]. The conversion of T_4_ into the metabolically active T_3_ ensures adequate energy metabolism to sustain milk production, even when TSH and T_4_ levels are relatively low. This lactation-driven metabolic reprogramming may represent an evolutionary adaptation in camels, allowing them to maintain lactation under the energy constraints of desert environments.

Age-related changes were also evident in thyrotroph morphology and thyroid hormone profiles. Younger camels (5–10 years) displayed higher T_3_ concentrations compared to older animals, consistent with the general decline in thyroidal activity with age documented in humans and livestock [29]. Morphologically, thyrotroph diameter, area, and nuclear dimensions were significantly greater in camels aged 11 years and above, suggesting a compensatory enlargement of cells despite lower functional output. This hypertrophy may reflect an attempt to sustain hormone production in the face of diminished secretory efficiency, a phenomenon also observed in aging pituitary tissues across mammalian species [30].

The interaction between lactation and age highlights an important feature of endocrine adaptation. While lactation in younger camels was clearly associated with elevated T_3_ and greater thyrotroph counts, this effect was less pronounced in older animals, where hormonal and morphological differences between lactating and non-lactating individuals were attenuated. Such reduced plasticity of the HPT axis with advancing age may contribute to the diminished reproductive and lactational performance of older female camels, as has been reported in other domestic ruminants [31]. The role of photoperiod and seasonal breeding patterns in camels may also influence thyrotroph activity, as seasonal modulation of pituitary function has been documented in other large herbivores [32].

Interestingly, the present findings contrast with those of Tajik et al., who reported no significant differences in T_3_ concentrations in dromedaries [33,34,35]. The increased thyrotroph counts in lactating females may reflect heightened demand for thyroidal stimulation, even if circulating TSH is reduced, highlighting the complex feedback loops governing the HPT axis.

The enlarged thyrotrophs and nuclei observed in older camels suggest an age-related decline in pituitary efficiency, whereby cellular hypertrophy attempts to compensate for reduced hormone output. Similarly, the heightened T_3_ concentrations and increased thyrotroph dimensions in males likely reflect their greater metabolic activity and potential seasonal endocrine modulation.

## 5. Conclusions

The present study demonstrates that lactation in camels is associated with a functional reorganization of the HPT axis, characterized by lower TSH and T_4_ but enhanced T_3_ availability. Age modifies these responses, with younger camels showing greater plasticity. These findings provide valuable insights into the endocrine strategies employed by camels to sustain lactation and adapt to age-related metabolic demands, and they align with the broader understanding of pituitary–thyroid interactions in mammals.

## Figures and Tables

**Table 1:** Mean adenohypophyseal thyrotroph morphometric parameters of lactating and non-lactating she camel (*Camelus dromedarius*) at different age groups (Immunocytochemistry).

Groups	5–10 years			11–Onward years			Overall Mean	
	Lactating	Non-Lactating	Overall Mean	Lactating	Non-Lactating	Overall Mean	Lactating	Non-Lactating
**Thyrotroph Count**	6.70 ± 0.2 ^a^	7.85 ± 0.07 ^a^	7.28 ± 0.39 ^A^	7.50 ± 0.33 ^a^	4.10 ± 0.19 ^b^	5.80 ± 0.33 ^B^	7.10 ± 0.21 ^A^	5.98 ± 0.48 ^B^
**Cell diameter (µm)**	11.37 ± 0.4 ^b^	14.41 ± 0.64 ^a^	12.90 ± 0.44	13.10 ± 0.55 ^a^	11.00 ± 0.28 ^b^	12.05 ± 0.42	12.23 ± 0.37	12.72 ± 0.48
**Cell area (µm^2^)**	106.10 ± 8.1 ^b^	141.71 ± 12.4 ^a^	139.57 ± 8.9	173.04 ± 13.5 ^a^	122.17 ± 8.30 ^b^	123.91 ± 7.72	137.83 ± 9.48	873.60 ± 94.07
**Cell volume (µm^3^)**	873.40 ± 94.07 ^c^	1830.20 ± 195.1 ^a^	1351.90 ± 1.4	1369.39 ± 188.8 ^b^	861.46 ± 116.4 ^c^	1115.42 ± 114.74	1121.49 ± 109.58	1345.83 ± 129.01
**Nucleus diameter (µm)**	3.57 ± 0.02 ^b^	5.10 ± 0.22 ^a^	4.30 ± 0.18	5.13 ± 0.31 ^a^	3.63 ± 0.16 ^b^	4.45 ± 0.20	4.35 ± 0.21	4.44 ± 0.16
**Nucleus area (µm^2^)**	10.87 ± 1.19 ^b^	21.55 ± 1.64 ^a^	16.21 ± 1.22	22.89 ± 2.61 ^a^	11.71 ± 0.91 ^b^	17.30 ± 1.55	11.88 ± 1.62	16.63 ± 1.13
**Nucleus volume (µm^3^)**	30.26 ± 5.04 ^b^	80.20 ± 8.54 ^a^	55.27 ± 5.99	93.83 ± 14.81 ^a^	32.14 ± 3.55 ^b^	62.99 ± 18.56	62.04 ± 8.79	56.17 ± 5.55

Means sharing similar letters (a, b, c) are not significantly different at *p* < 0.05. A, B mean values bearing superscripts in the same row differ significantly (*p* < 0.05)

## Data Availability

The relevant data are provided in the paper. The data of the current experiment can be obtained from corresponding author when needed.
